# Supplementary material for: Plasmodium DNA-mediated TLR9 activation of T-bet+ B cells contributes to autoimmune anaemia during malaria
Source: Nat Commun. 2017 Nov 3;8:1282. doi: 10.1038/s41467-017-01476-6 (PMC5670202; doi:10.1038/s41467-017-01476-6)
Supplement: Supplementary file 1 — Supplementary Information [file 41467_2017_1476_MOESM1_ESM.pdf]

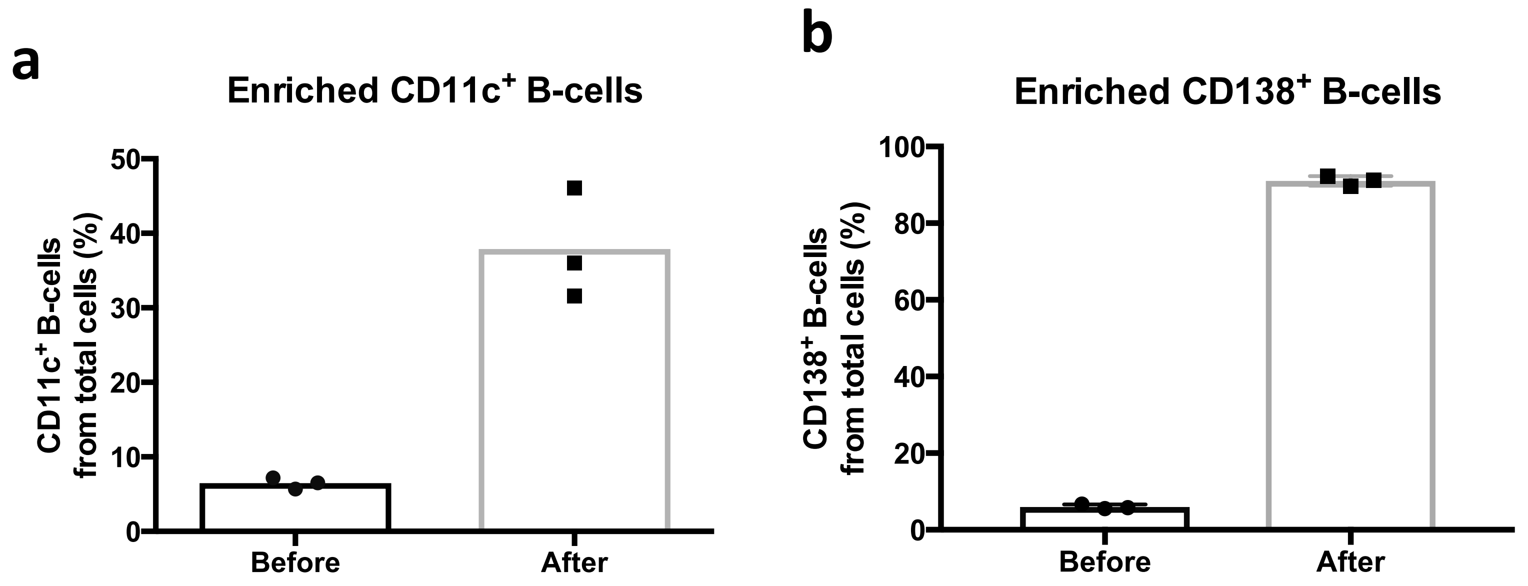

Fig. S1. Enrichment of CD11c$^+$ B-cells and CD138$^+$ plasma. Related to Figure 2. Percentage of CD11c$^+$ B-cells (CD19$^+$) (A) and CD138$^+$ plasma cells (B) from *P. yoelii*-infected mice before and after enrichment with antibody coated-magnetic beads. Purification was assessed by flow cytometry. Bars represent the means ± SD of n=3 mice.

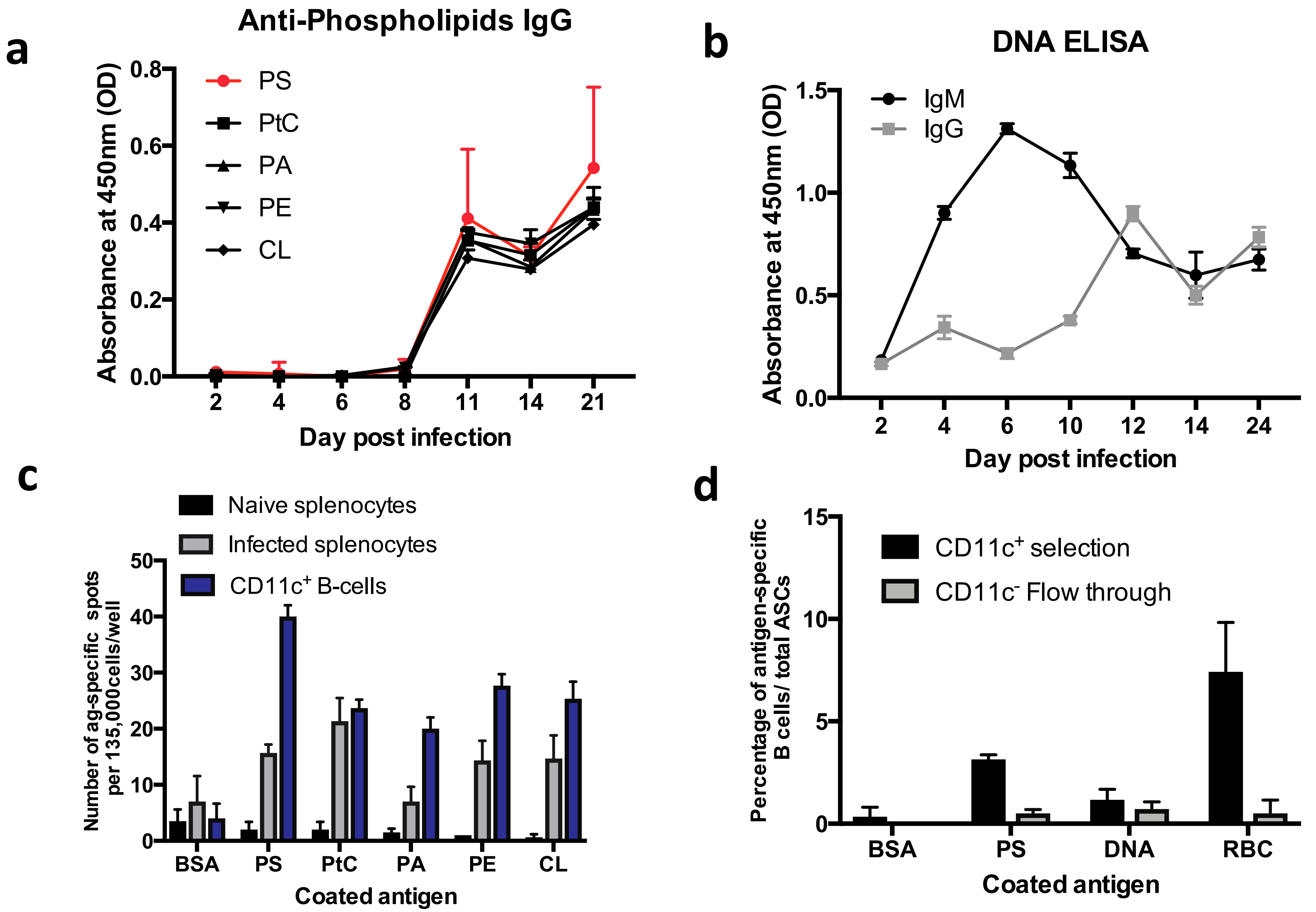

Fig S2. ***P. yoelii*-infected mice develop different kinds of autoantibodies. Related to Figure 2.** ELISA (a and b) and ELISpot (c and d)for anti-phospholipid (a and c) and anti-dsDNA (b and d)antibodies in plasma (a and b) or from Day 8 pi splenocytes or CD11c^{+} cells(c and d) from *P. yoelii*-infected Swiss Webster mice or uninfected mice splenocytes. Bars graphs represent the mean ± SD of n=3 mice. Analyzed by Student unpaired t-test *$p < 0.05$. PS=phosphatidylserine, PtC= phosphatidylcholine, PA= Phosphatic Acid, PE= Phosphatidylethanolamine, CL= Cardiolipin

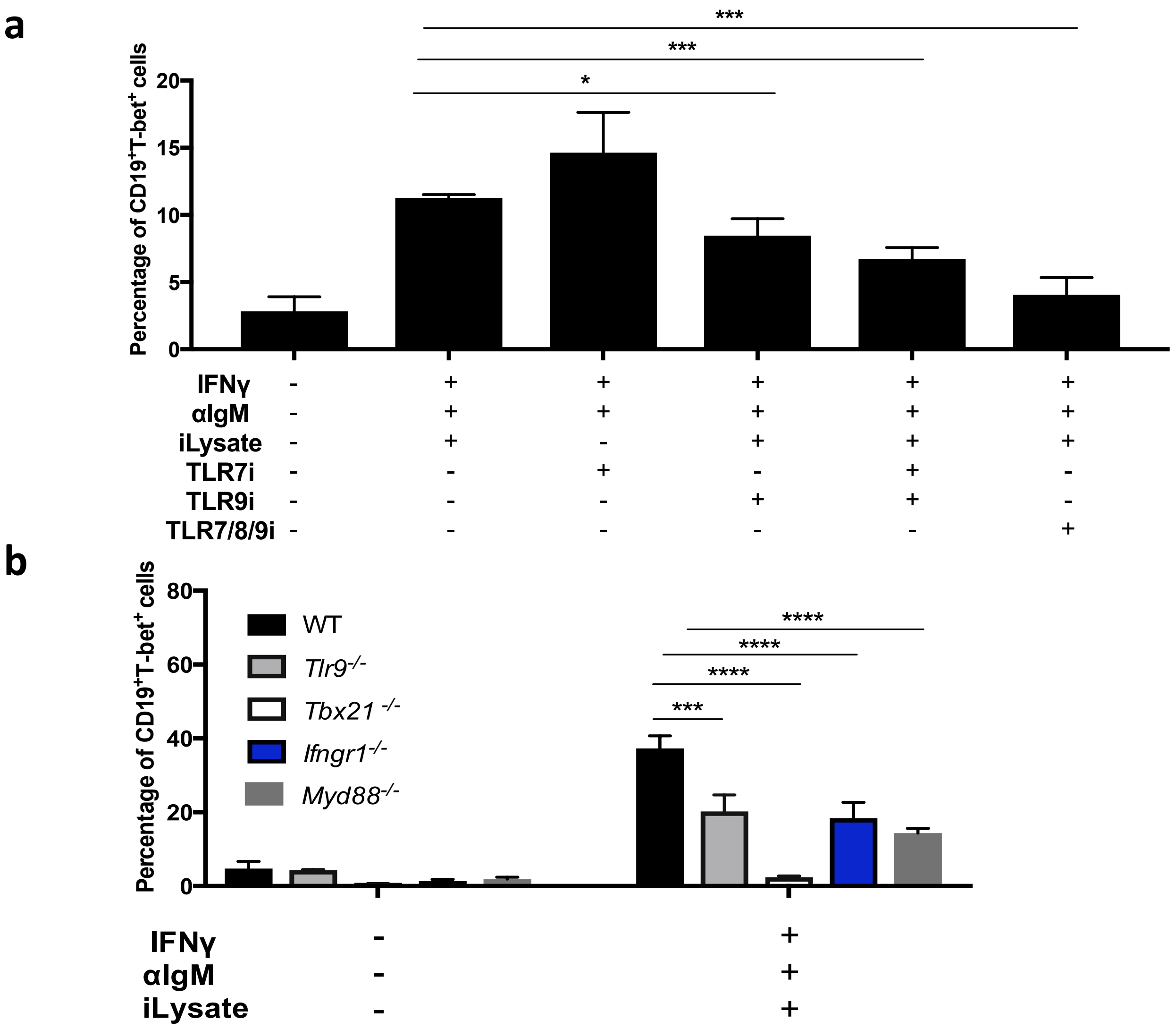

Fig S3. Effect of Myd88 and TLR7, 8 and 9 inhibition in *Plasmodium*-induced T-bet expression on B-cells . Related to Figure 4. Purified naïve B-cells ($CD43^-$) from uninfected mice were cultured under the indicated conditions for 3 days, when T-bet expression was determined in $CD19^+$ cells. (a) Cells were incubated in the presence of IFNγ, anti-mouse IgM and *P. yoelii*-infected erythrocyte lysate (iLysate) with the addition of antagonists of TLR7 or TLR9 (ODN 20958 TLR7i or A151 TLR9i), or with antagonist for TLR7,8 and 9 (ODN 2088 TLR7/8/9i). (b) Cells from uninfected WT or from *Ifngr1*$^{-/-}$,*Tbx21*$^{-/-}$, *Tlr9*$^{-/-}$ or *Myd88*$^{-/-}$ mice were incubated or not with IFNγ, anti-mouse IgM and *P. yoelii*-iRBC lysate (iLysate). Bars represent the means ± SD of n=3 mice. Significance determined by one-way ANOVA *$p < 0.05$, ***$p < 0.001$, ****$p < 0.0001$.

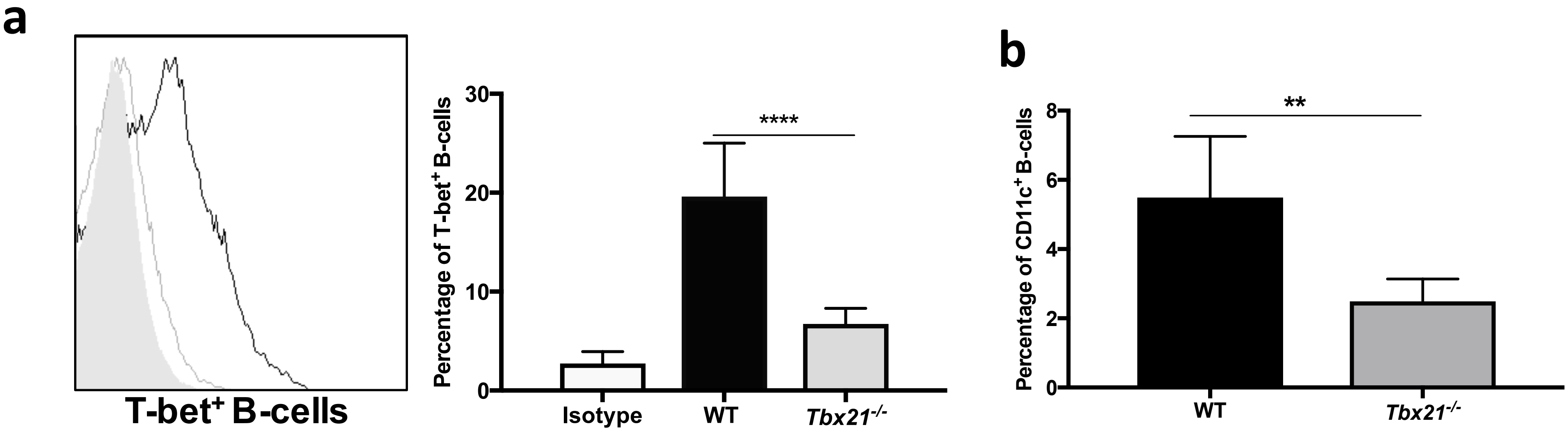

Fig. S4. Control staining for *Tbx21$^{-/-}$ P.yoelii*-infected mice. Related to Figure 5. (A and B) Histogram profile (Isotype control gray shadow, WT black line, gray line *Tbx21$^{-/-}$*) and quantification in percentage of T-bet $^{+}$ (A) or CD11c^{+} (B) expression from B-cells (CD19^{+}) from either *P. yoelii*-infected WT or *Tbx21$^{-/-}$*. Bars graphs represent the mean ± SD of triplicates of n=2 mice. **$p < 0.01$,***$p < 0.001$.

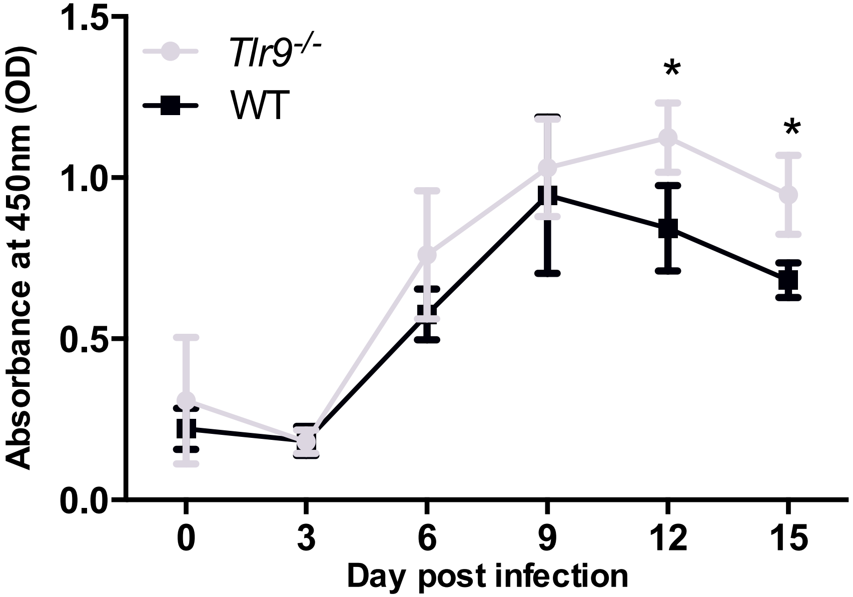

Fig S5. ***Tlr9$^{-/-}$*** **mice infected with** ***P. yoelii*** **develop greater general autoimmunity. Related to Figure 7.** ELISA for anti-dsDNA antibodies in plasma from *P. yoelii*-infected WT (black line) or *Tlr9$^{-/-}$* mice (gray line). Bars graphs represent the mean ± SD of n=3 mice. Analyzed by Student unpaired t-test $^{*}p < 0.05$.

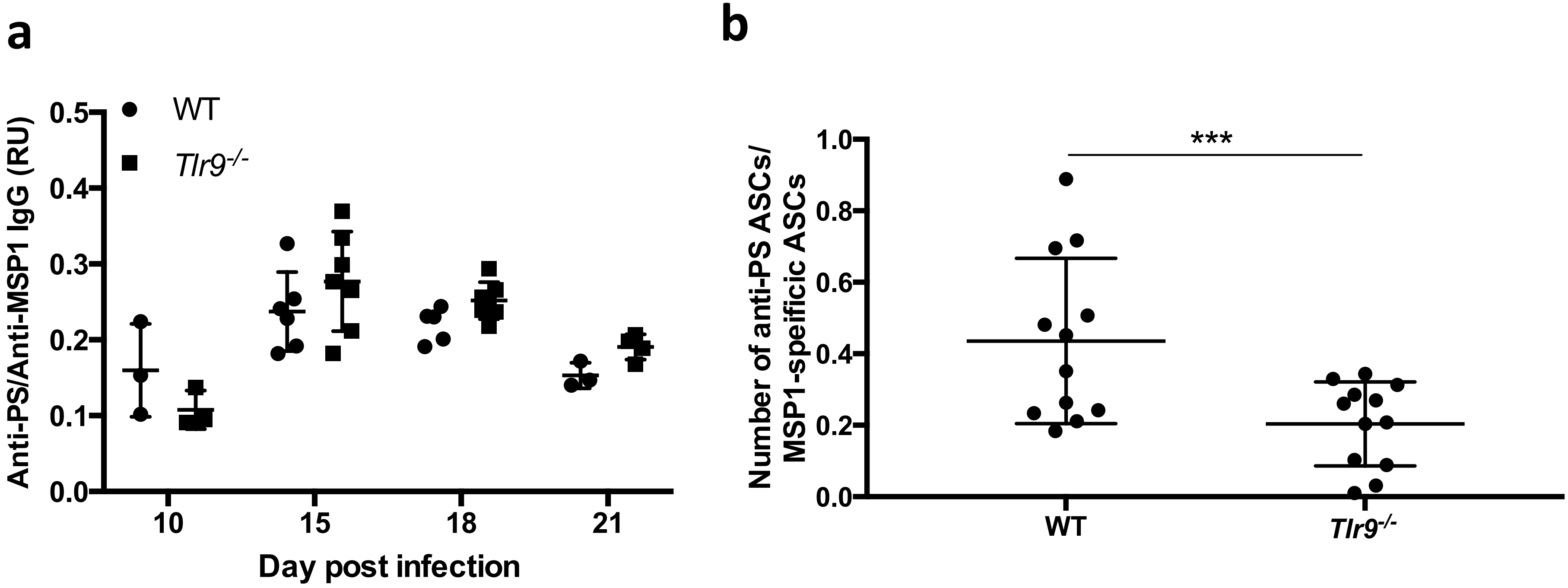

Figure S6. B-cell specific deletion of TLR9 leads to a reduced anti-PS B-cell response during *P. yoelii* infection. Related to Figure 7. (a) Quantification of the ratio of anti-PS IgG versus anti- *P.yoelii* MSP1 antibodies (RU, Relative Units) from B-cell-specific chimeras reconstituted with either WT (circle) or *Tlr9*$^{-/-}$ B cells (squares). (b) Number of antigen-specific spots for PS over parasite *P.yoelii* MSP1 of B-cell specific *Tlr9*$^{-/-}$ chimeric mice at D.18 p.i. Significance determined by unpaired Student's *t* test. Graphs represent the individual values of n=11 mice (a) or n=4 mice (b) ***p < 0.001
